# Modulation of lipid biosynthesis contributes to stress resistance and longevity of *C. elegans* mutants

**DOI:** 10.18632/aging.100275

**Published:** 2011-02-25

**Authors:** Robert J. Shmookler Reis, Lulu Xu, Hoonyong Lee, Minho Chae, John J. Thaden, Puneet Bharill, Cagdas Tazearslan, Eric Siegel, Ramani Alla, Piotr Zimniak, Srinivas Ayyadevara

**Affiliations:** ^1^ Central Arkansas Veterans Healthcare Service, Little Rock, AR 72205, USA; ^2^ Department of Geriatrics, University of Arkansas for Medical Sciences, Little Rock, AR 72205, USA; ^3^ Department of Biochemistry and Molecular Biology, University of Arkansas for Medical Sciences, Little Rock, AR 72205, USA; ^4^ Department of Biostatistics University of Arkansas for Medical Sciences, Little Rock, AR 72205, USA; ^5^ Department of Pharmacology and Toxicology, University of Arkansas for Medical Sciences, Little Rock, AR 72205, USA

**Keywords:** Aging, longevity, lipid (Fatty Acid), lipoperoxidation, nematode, RNAi

## Abstract

Many lifespan-modulating genes are involved in either generation of oxidative substrates and end-products, or their detoxification and removal. Among such metabolites, only lipoperoxides have the ability to produce free-radical chain reactions. For this study, fatty-acid profiles were compared across a panel of C. elegans mutants that span a tenfold range of longevities in a uniform genetic background. Two lipid structural properties correlated extremely well with lifespan in these worms: fatty-acid chain length and susceptibility to oxidation both decreased sharply in the longest-lived mutants (affecting the insulinlike-signaling pathway). This suggested a functional model in which longevity benefits from a reduction in lipid peroxidation substrates, offset by a coordinate decline in fatty-acid chain length to maintain membrane fluidity. This model was tested by disrupting the underlying steps in lipid biosynthesis, using RNAi knockdown to deplete transcripts of genes involved in fatty-acid metabolism. These interventions produced effects on longevity that were fully consistent with the functions and abundances of their products. Most knockdowns also produced concordant effects on survival of hydrogen peroxide stress, which can trigger lipoperoxide chain reactions.

## INTRODUCTION

The first mutation observed to extend lifespan in the nematode *Caenorhabditis elegans*, *age-1*, was discovered through a screen for increased longevity among progeny exposed to chemical mutagenesis [1]. A decade later, mutations in the *daf-2* gene (then known only to function in developmental progression) were found to double the normal lifespan [2], and both genes were subsequently shown to function in the insulin-like signaling pathway [3,4]. Many lifespan-augmenting mutations have since been isolated, chiefly in *C.elegans*, but also in yeast, fruit flies and mice. The most pronounced longevity effects of single-gene mutations have generally been seen in *C. elegans*, in which the disruption of several dozen genes has been reported to prolong lifespan by 50-150% [2,5]. We recently showed that two *age-1* nonsense mutations (*mg44* and *m333*), previously observed to increase longevity by 2- to 2.5-fold in the first generation of homozygotes for either mutation [5,6], extend adult lifespan nearly tenfold in their offspring [7].

Although many life-prolonging mutations have been reported, these were generated in diverse genetic backgrounds that are also likely to influence longevity. Moreover, life extension for mutant strains has been measured relative to wild-type control stocks that also varied in longevity among studies, and even within a study were not always isogenic with the mutations. As an example of genetic variation among ostensibly identical control stocks, lifespans were found to range from 12 to 17 days among six common laboratory stocks of the Bristol-N2 wild-type strain, which have been widely used as controls in survival studies [8]. In order to remove many secondary genetic variations not tightly linked to the mutations identified as affecting life span (which could confound the results with respect to other endpoints), we constructed a set of near-isogenic longevity mutants. Each mutant line was outcrossed for at least six generations to an “N2 male” strain from the Riddle laboratory (Caenorhabditis Genetics Center; here designated N2DRM), the longest-lived of six N2 stocks compared in a single study [8]. Simultaneous survivals were then conducted for multiple mutants, in parallel with their isogenic wild-type controls.

Lipid reserves, quantified in diverse ways, have been implicated in nematode lifespan [9-14] and innate immunity [11]. This area is not without controversy, however, since various measures of lipid content have produced quite distinct results. For example, several long-lived mutant strains were found to have markedly elevated lipid stores, as monitored by Sudan Black staining [4], whereas both dietary restriction and metformin increase lifespan while *reducing* Nile-Red-stained lipid stores [15]. Such apparent contradictions, as well as the poor correlation between lifespan and triacylglyceride storage among long-lived *daf-2* alleles [9], suggest that lipid levels *per se* play no consistent, causal role in longevity. GC-MS quantitation of lipids in *C. elegans* appears to correlate best with major lipid repositories stained by Oil Red O [16]. In any case, lipid synthesis, transport, and utilization may have consequences for health and longevity that are largely independent of their net impact on the size of lipid stores [17]. Structural properties of fatty acids, such as chain length, branching, or unsaturated sites, have received little prior attention as possible determinants of nematode lifespan.

In the present study, we utilized gas chromatography coupled to mass spectrometry (GC-MS) to analyze fatty-acid composition across ten *C. elegans* strains of nearly identical genetic background, and the observed trends were corroborated by transcript-level changes observed for genes involved in fatty acid biosynthesis. Those trends are largely dominated by the longest-lived strains, all of which carry mutations in insulin/IGF-1 signaling, thus constraining our conclusions to that pathway. Nevertheless, RNAi knock-down of the same genes in wild-type worms had effects on survival of an oxidative stress, and on lifespan, that are very largely consistent with predictions based on abundance trends. Those results strongly imply that the same structural properties of lipids implicated from the mutant panel (chain length, degree of saturation) also play a distinct causal role in lifespan determination, even in worms with normal insulinlike signaling.

## RESULTS

### Construction and characterization of outcrossed strains

**Figure 1.**
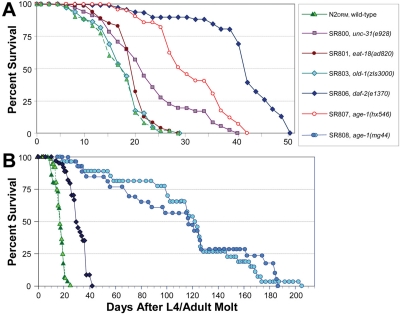
Survivals on agar of wild-type and near-isogenic mutant strains. Worm cohorts were syn-chronized by recovery of eggs after lysis of gravid hermaphro-dites in alkaline hypochlorite (see Materials and Methods), and then by manual selection of L4 larvae. Groups of 25 adults (50 total per group) were transferred to fresh plates daily until no further progeny were produced, and on alternate days thereafter. The x-axis indicates adult age in days, thus excluding effects of slowed development. **A** illustrates examples of replicate experiments, all of which produced similar sets of survival curves. **B** shows survival data for N2DRM, *daf-2(e1370)* and *age-1(mg44)-F2* worms, combined with earlier data (dark green triangles and light blue circles, redrawn from [7].

A series of congenic *C. elegans* longevity-mutant strains was created by repeated out-crossing of strains that had been reported to be long-lived, although they were initially isolated in diverse genetic backgrounds and characterized relative to different N2 control stocks [3,7,18-20]. Survivals were conducted for these strains (e.g., Figure 1); median and mean lifespans from several assays, normalized to their isogenic control (N2DRM), are summarized in Table 1. Adult survival times for these strains varied slightly between experiments, whereas relative lifespans (normalized to wild-type controls, assessed simultaneously) were highly reproducible. Several departures from the literature values were observed, which could be due to strain outcrossing into the N2DRM genetic back-ground, use of a different “wild-type” reference strain than originally employed to evaluate life extension, or inter-laboratory differences in culture conditions. Among the strains utilized herein, *unc-31(e928)* and *eat-18(ad820)* conferred less life extension than previously reported, and strain SR803 (carrying the *old-1(zIs3000)* transgene integrated in an N2DRM background [21]) was indistinguishable from N2DRM controls.

**Table 1.** *C. elegans* near-isogenic mutant strains used in lipid studies.

**Strain**	**Genotype**	**Median Life span Relative to N2DRM Mean (Range)**	**Mean Life span Relative to N2DRM Mean (Range)**	**Affected Protein(s)**	**Phenotype**
N2DRM	wild type	**1.00** (0.94 - 1.06)	**1.00** (0.94 - 1.06)	None	Normal control, isogenic to all mutant strains
SR803	*old-1(zIs3000)*	**1.03** (1.03-1.03)	**1.04** (1.03-1.05)	FGF/PDGF-related receptor kinase	Integrated transgene reported to confer long-lived phenotype
SR801	*eat-18(ad820)*	**1.14** (1.14-1.14)	**1.10** (1.07-1.12)	Novel transmembrane protein (modulator of pharyngeal pumping)	Long-lived, probably via DR
SR800	*unc-31(e928)*	**1.21** (1.14-1.27)	**1.35** (1.34-1.36)	CAPS ortholog (Ca^++^-dependent activator of neurosecretion)	Long-lived, probably via DR
SR806	*daf-2(e1370)*	**2.26** (2.26-2.26)	**2.12** (2.07-2.16)	IGF/Insulin-like receptor^ts hypomorph^	Long-lived, via IIS disruption
SR818	*daf-16(m26); daf-2(e1370)*	**0.95** (0.85-1.05)	**0.94** (0.90-0.98)	IGF/Ins-R^ts hypomorph^ + FOXO^hypomorph^	Reversion control for SR806
SR807	*age-1(hx546)*	**1.63** (1.43-1.81)	**1.63** (1.60-1.66)	PI3K^ts hypomorph^	Long-lived, via IIS disruption
SR819	*daf-16(m26); age-1(hx546)*	**1.06** (1.06-1.06)	**1.07** (1.03-1.11)	PI3K^ts hypomorph^ + FOXO^hypomorph^	Reversion control for SR807
SR808	*age-1(mg44)*	**9.6** (9.2-9.9)*	**9.5** (8.5-10.5)*	PI3K^null^	Long-lived, via IIS and other signaling pathways
SR820	*daf-16(m26); age-1(mg44)*	**1.06** (1.06-1.06)	**1.10** (1.07-1.12)	PI3K^null^ + FOXO^hypomorph^	Reversion control for SR808

Life spans were determined at least twice, for independent expansions, with 35 - 50 worms per group (20 - 38 uncensored deaths). Further details are provided in Supplemental Table S1. The age-1 gene encodes the catalytic (p110ɑ) subunit of phosphatidyl-inositol 3-kinase (PI3K); the *daf-2* gene encodes a cell membrane receptor for insulin-like ligands. The *daf-16* gene encodes a FOXO transcription factor, phosphorylated via the DAF-2/AGE-1/PDK-1/AKT kinase cascade. The life-extension phenotypes of *age-1* and *daf-2* are largely or entirely reversed by double mutations with daf-16 [5,7]. *Survival data for the F2 generation of SR808 [*age-1(mg44)*] are from reference 7, not repeated here.

The wild-type progenitor strain N2DRM, near-isogenic with all mutant strains studied, served as their common control. Double mutants with *daf-16(mu26)* provided additional controls that largely or entirely revert life-span and stress-resistance traits of single-gene insulin/IGF-1 signaling (IIS) mutants [7,22,23], although it cannot be assumed that all affected traits are identically reversed. By this comparative design, we sought features of fatty-acid metabolism that correlate with strain longevity, while controlling for mutation-specific phenotypes not associated with longevity. Because all three double-mutant controls and one of the reportedly long-lived mutants, *old-1(zIs3000)*, had longevities very close to that of N2DRM controls, in log-linear regressions they serve chiefly as additional controls for the consequences of the more effective IIS mutants. Although relatively modest, the lifespan extensions by *unc-31* and *eat-18* mutations were reproducible and sometimes significant; they thus provide informative assay points that contribute to all measures of correlation, and in particular to rank-order correlations.

### Identification and quantitation of fatty-acid chains

In total, 23 fatty acids (FA) were detected, positively identified, and quantified for the worm samples examined, which included most of the fatty acids previously reported for wild-type *C. elegans* [24,25]. These comprised two cyclopropane fatty acids (9,10-methylene 16:0 and 11,12-methylene 18:0), two monomethyl branched-chain fatty acids (15:0-*iso* and 17:0-*iso*), five saturated FAs (14:0, 16:0, 18:0, 20:0 and 22:0), five mono-unsaturated fatty acids (16:1∆9, 18:1∆9, 18:1∆11, 20:1∆11, and 22:1∆13), and eight poly-unsaturated fatty acids, of which five are n-6 (or “omega-6”) fatty acids (18:2∆9,12, 18:3∆6,9,12, 20:3∆8,11,14, 20:4∆5,8,11,14, and 20:2∆11,14), while three are n-3 or “omega-3” chains (18:3∆9,12,15, 20:4∆8,11,14,17, and 20:5∆5,8,11,14,17). Of these, fatty acids 20:0, 22:0, 20:2∆11,14(n-6) and 22:1∆13(n-9) do not appear to have been previously reported in this species [24,25]. The first three are presumed to be +2C elongation products of the fatty acids 18:0, 20:0 and 18:2∆9,12(n-6), respectively, whereas 22:1(n-9) would require two +2C additions to 18:1∆9(n-9). The fatty acid 18:4(n-3), shown in schemas as a possible intermediate to formation of 20:4(n-3), was not observed; it has been observed in *elo-1* mutants but not in worms with normal lipogenesis [25,26]. Supplemental Table S3a lists the percent content by strain, of 19 of the above fatty acids, excluding several that were less reliably identified or quantified in one or more strains.

The omega-terminal branched-chain fatty acids 15:0-*iso* and 17:0-*iso* were found in all *C. elegans* mutant and control strains. These odd-carbon-number fatty acids are produced in *C. elegans* by de novo synthesis [27,28], by ELO-5 and ELO-6 extension of branched primers, with essentially none (<1%) arising from ingested bacteria [25]. In contrast, fatty acids containing a cyclopropane ring are believed to be entirely of bacterial origin and vary in quantity with the bacterial diet [29]; they were not included in the analyses described. However, because total cyclo-propane fatty acids varied rather little among strains (6.3 -10.6% of total lipids), their inclusion or exclusion had very little effect on the percentages calculated for other FAs.

**Figure 2.**
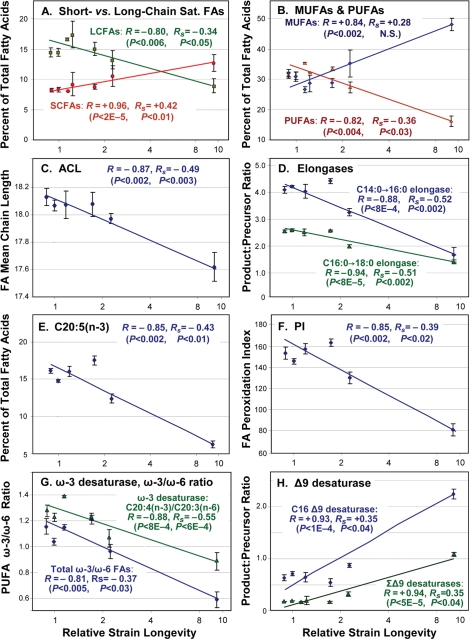
Trends of fatty-acid composition with increasing life span. In each panel, data points with error bars indicate means ± standard errors, of GC-MS quantitations for the indicated lipid component expressed as a percent of total fatty acids quantified for that run. *X*-axes display median lifespans from Table 1: [strains; number of independent cohorts/runs]: **1.0** [N2DRM, SR803, SR818, SR819, SR820; *n* = 20], **1.1 - 1.2** [SR800, SR801; *n* = 4], **1.6** [SR807; *n* = 5], **2.3** [SR806; *n* = 3] and **9.6** [SR808; *n* = 4]. Medians (Table 1) are more robust than means to effects of outliers. Y-axes show (panels **A**, **B** and **E**) the percent of total fatty acids in each indicated class; (**C**) Average Chain Length; (**D**) activities of fatty-acid elongases, inferred from FA ratios (C16:0/C14:0, C18:0/C16:0); (**F**) Peroxidation Index (defined in Table 2 legend); (**G**) activities of ω-3 desaturases, inferred from FA ratios (e.g., C20:4(n-3)/C20:3(n-6)), and ω-3/ω-6 ratios for all PUFAs; and (**H**) inferred Δ9 desaturase activities (C16:1Δ9/C16:0, C18:1Δ9/C18:0). In each panel, *R* denotes the Pearson correlation coefficient, reflecting a linear relationship between the log_(10)_ of strain lifespan and the indicated fatty-acid parameter. *R_S_* is the Spearman correlation coefficient, relating the rank-order of strain lifespan to rank-order of fatty-acid parameter per class (e.g., the % of total FAs) in 36 assays. Table 2 lists the corresponding significance thresholds. *R* values shown here were calculated by treating each of 10 strains as one sample point. This reduces significance (due to lower *N*), but increases *R,* relative to points derived from the 36 GC-MS measurements on independent cohorts, due to the lower variance of means.

Quantitations of fatty acid classes for most of the congenic strains are summarized in Table 2. Double-mutant controls, in which *daf-2* and *age-1* mutants are reverted by a *daf-16* mutation [7,22,23], agreed well with other “normal-lifespan” controls and thus were omitted from this table and Figure 2, although they contributed to the correlations. Columns at the right indicate the strengths of linear (Pearson) and rank-order (Spearman) correlations with the logarithm of strain longevity. Several of the strongest correlations are shown graphically in Figure 2, in which the mean percent of total fatty acids in each class was plotted as a function of the log_(10)_ of strain lifespan. It is apparent from this figure that most log-linear correlations are dominated by the three longest-lived mutants: *age-1(hx546), daf-2(e1370)* and *age-1(mg44)*. Because these three mutations affect components of the insulin/IGF-1 signaling (IIS) pathway, no conclusions based on correlation analyses alone will necessarily apply outside that pathway. Nevertheless, they serve to dissect the properties of IIS attenuation that bear most directly on longevity, from those that are peculiar to specific gene mutations or to other sequence changes tightly linked to an identified mutation.

**Table 2.** Fatty acid composition of isogenic *C. elegans* strains of varying lifespan (*N.B.: double-mutant control strains had values close to N2, and are not shown here although included for calculation of correlation coefficients.*)

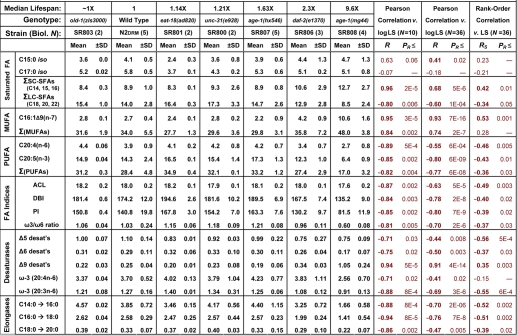

FA amounts are indicated as percents of total FAs. Longevity was assessed at least twice per strain, with the results as indicated in Table 1, column 3. For rank-order correlations, strains with median lifespans near the value of wild-type N2DRM (relative median lifespans near 1), were assigned the same longevity rank, since they could not be ordered unambiguously with respect to one another. These strains were the control strain N2DRM, the double-mutant control strains SR818 [*daf-16(m26); daf-2(e1370)*], SR819 [*daf-16(m26); age-1(hx546)*] and SR820 [*daf-16(m26); age-1(mg44)*], and SR803 [*old-1(zls3000)*]. In these analyses, *N* is the total number of biological replicates. For each correlation coefficient *R*, calculated from *N* data points, its *P* value was ascertained from its *t*-transformation, *t* = |*R*| · [(*N* – 2) / (1 – *R*^2^)]^½^, treating the result as coming from a *t* distribution with *N*–2 degrees of freedom.

Average Chain Length, ACL=[(Σ[%14C]×14) + (Σ[%15C]×15) + (Σ[%16C]×16) + (Σ[%17C]×17) + (Σ[%18C]×18) + (Σ[%20C]×20) + (Σ[%22C]×22)]/100, where sums Σ are for all fatty acid chains of the indicated length (14C, 15C, 16C etc.).

(Σ[%22C]×22)]/100, where sums Σ are for all fatty acid chains of the indicated length (14C, 15C, 16C etc.).

Double Bond Index, DBI = (Σ[%*n*C:1] × 1) + (Σ[%*n*C:2] × 2) + (Σ[%*n*C:3] × 3) + (Σ[%*n*C:4] × 4) + (Σ[%*n*C:5] × 5), where Σ is for *n*=12–22.

Peroxidation Index, PI = (Σ[% *n*C:1] × 0.025) + (Σ[% *n*C:2] × 1) + (Σ[% *n*C:3] × 2) + (Σ[% *n*C:4] × 4) + (Σ[% *n*C:5] × 6), taking Σ for *n*=12–22.

The first correlation column, headed “*N*=10”, treats each of the ten strains as a single data point, defined by the means of its lifespan and GC-MS measurements. The other two correlation columns (“*N*=36”), consider each GC-MS measure on an independent cohort of nematodes as one data point, since these lipid quantitations had far higher variance than did relative lifespan. Rank-order correlations, shown in the right-most column, are independent of all assumptions regarding data distribution or transformation, and are also conservative in that they are influenced equally by strains of moderate or extreme longevity. To the extent that a proximal factor impacting lifespan is expected to vary in abundance in proportion to longevity, however, Pearson log-linear correlations have the merit of preserving that information. Of 21 fatty-acid parameters quantified in Table 2, 19 had Pearson log-linear correlations that were nominally significant at *P*<0.05, and 13 were highly significant at *P*<0.0024 (i.e. <0.05/21). This was true whether 10 or 36 data points are considered. In the description that follows, *R* and *P* values are given only for 10-point regressions, to maintain consistency with Figure 2. We note, however, that the 36-point analyses better reflect the extent of uncertainty due to biological variation within each group; they usually result in lower correlation coefficients, but improved significance.

Seventeen of these longevity-dependent trends met a more stringent criterion of having nominally significant Spearman rank-order correlation coefficients, *P*<0.05, of which seven were highly significant at *P*<0.0024. The data of Table 2 were compiled from a more complete listing that comprises quantitative data for 19 FA species (Supplemental Table S3a) and 18 derived measures of biological interest (Table S3b).

A positive correlation was observed between longevity and the abundance of short-chain saturated fatty acids C14:0, C16:0 and C15*-iso*, a trend that was reversed for the longer-chain classes 18:0, 20:0 and 22:0 (Figure 2A and Table 2). The same pattern was repeated among the monounsaturated fatty acids (MUFAs, Figure 2B), with positive correlations to lifespan for C16:1∆9 and C18:1∆11, but weaker negative correlations for C20:1 and C22:1 (Tables 2 and S3a). As expected from these trends in individual lipid species, the average chain length of all fatty acids declined in association with lifespan (Figure 2C), with log-linear correlation coefficient *R* = −0.87 (*P*< 0.002), and rank-order *R_S_* = −0.49 (*P*<0.003).

Of five major polyunsaturated fatty acid (PUFA) species observed, four fell significantly in amount as longevity increased: 18:3(n-6), 20:4(n-6), 20:4(n-3) and 20:5(n-3), whereas 20:3(n-6) did not (Figure 2, B and E; Tables 2 and S3). Unlike mammals, which require dietary PUFAs to maintain health, nematodes possess all of the enzymes necessary for their biosynthesis, and thus their lipid composition depends on genetics and gene-expression as well as diet (see below). Although it remains controversial whether lifespan is indeed limited by reactive oxygen species [31,32], there is broad agreement that lipoperoxidation products can amplify via chain reactions [32,33], and thus pose a serious biological hazard for all cells. In PUFAs, the carbons situated between double bonds are especially vulnerable to peroxidation, whereas saturated and monounsaturated chains are several hundredfold less susceptible [34]. The Double Bond Index (DBI) is a simple weighted average of the number of double bonds per fatty acid molecule: Σ (1·Σ(1-DB %) + 2·Σ(2-DB %) + …. + 5·Σ(5-DB %) [35]. The Peroxidation Index (PI) is calculated in similar fashion, except that the contributions of MUFAs and PUFAs are weighted to reflect their susceptibilities to lipoperoxidation [34].

DBI and PI displayed quite similar monotonic declines with longevity, giving correlation coefficients of −0.84 and −0.85 (each *P*<0.0024) and rank-order correlation coefficients of −0.40 and −0.39 (*P*<0.02; Table 2 and Figure 2F).

*C. elegans*, unlike mammals, possesses omega-3 (i.e., n-3) desaturase enzymes. They are thus able to convert arachidonic acid, 20:4∆5,8,11,14(n-6), to eicosapen-taenoic acid (EPA), 20:5∆5,8,11,14,17(n-3), and the level of ω-3 desaturase activity can be inferred from the ratio of those chains [30]. Estimated ω-3 desaturase activity declined with lifespan (Figure 2G and Table 2), with log-linear correlation coefficients *R* of −0.71 (*P*<0.02) and −0.88 (*P*<10^−3^). Among all PUFAs, the ratio of ω-6 to ω-3 fatty acids also declined (Figure 2G) with a log-linear correlation coefficient of −0.81 (*P*< 0.005). Although many aspects of human health are affected by dietary intake of ω-3 fatty acids, and tend to benefit from reduction of our high ω-6/ω-3 intake ratio, this reflects constraints on mammalian biosynthesis of lipids and of lipid-derived eicosanoid signaling molecules, rather than lipoperoxidation potential (which is somewhat lower for ω-6 than ω-3 fatty acids [34]). Because the same constraints do not apply to nematode biosynthesis of lipids [25], the impact of the ω-6/ω-3 ratio on *C. elegans* health is not known.

The levels of other desaturase activities can be inferred in like manner from their product/substrate ratios [30]. Based on these ratios (Tables 2 and S3b), the imputed levels of Δ6 desaturase showed a moderate inverse correlation to longevity (*R*= −0.75, *P*<0.02), whereas two ratios indicative of Δ9 desaturase activities (16:1Δ9/16:0 and 18:1Δ9/18:0) displayed strongly positive correlations with lifespan (Figure 2H), with a combined log-linear *R* value of +0.94 (*P*<5×10^−5^). These results are fully consistent with a study of the effects of mutations to Δ9 desaturases, encoded by *fat-5, -6* and *-7* genes in *C. elegans* [24]. The Δ9 desaturases are responsible for the initial introduction of a double-bond in a saturated fatty acid chain, and are thought to serve as “pacemaker” enzymes for lipid desaturation pathways [24,25]. It is thus paradoxical that the longest-lived mutant strains had the highest apparent Δ9 desaturase activity (Figure 2H) and the highest amounts of both 16:1Δ9 and 18:1Δ9 (Table S3), and yet the lowest PUFA levels (Figure 2B). This implies that additional regulation must occur downstream of the Δ9 desaturases, to favor or impede further desaturation. The declines with increasing lifespan, seen here for Δ6 and ω-3 desaturases (Table 2), support that interpretation.

Because the longest-lived strain, SR808, differs from the other strains with respect to fertility (*age-1(mg44)*-F2 homozygotes being completely infertile [7]), we were concerned that the longevity trends observed in FA content may have been confounded by variation in quantities of egg lipid stores. We therefore ran two post-hoc comparisons by ANOVA. In the first, three control strains were analyzed for FA content, comparing them at day 3 (egg-laying adults) vs. day 6 of adulthood, by which time they were post-gravid, pre-senescent adults, devoid of internal eggs or embryos by microscopic examination. None of the 20 FA measures showed a significant effect of eggs that could have contributed to the observed trends with longevity. The effect of infertility would often have opposed the shift seen in the long-lived SR808 [*age-1(mg44)*] strain, but in those instances where it could have contributed to that strain's exceptional FA content, the estimated effect size ranged from 3-26% of SR808's shift (data not shown). The second comparison simply re-evaluated the association between lifespan and FA content, considering only postgravid samples from four strains plus nongravid *age-1(mg44)*-F2 samples (SR808). The resulting correlations, summarized in the two right-hand columns of Table 3, closely mirrored the overall trends with lifespan observed previously (columns 2 and 3, taken from Table S3). Although the nongravid analyses carried less statistical significance, as expected in view of their much smaller sample size, the correlation coefficients for these 12 parameters were either similar to, or greater than, the corresponding coefficients from the full data set. The observed longevity trends thus cannot be attributed to strain differences in their egg or embryo content.

**Table 3.** Fatty acid trends with lifespan are not attributable to gravidity differences

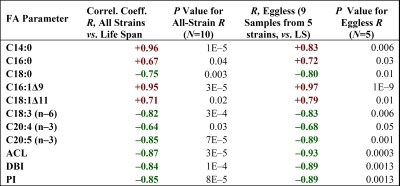

Data in columns 2 and 3 are taken from Supplemental Table S3.

### Transcriptional regulation of lipid biosynthesis

**Figure 3.**
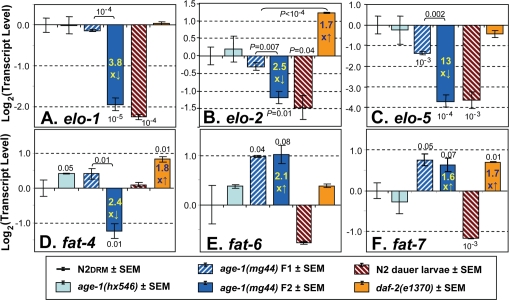
Steady-state transcript levels for genes of lipid metabolism. Real-Time Polymerase Chain Reaction (RT-PCR) was performed for the six indicated groups of *C. elegans* (see legend panel). In the experiment shown, the number of independent biological preparations was as follows (groups as displayed from left to right): N2DRM adults, *N*=8; SR807 [*age-1(hx546)*]*, N*=3; SR808 [*age-1(mg44)*], first (F1) homozygous generation*,N*=3; SR808 [*age-1(mg44)*], F2 generation*,N*=4; N2DRM dauer larvae, *N*=2; and SR806 [*daf-2(e1370)*], *N*=3. The ordinate (*y* axis) displays the base-2 logarithm of transcript level, after normalization to N2DRM controls (which thus always have a mean log_2_ value of zero). The *y* value is equivalent to - [t_c_(mutant) - t_c_(N2)], where t_c_ is the cycle number required to achieve an arbitrary threshold level of PCR amplification product. *P* values of <0.1 (based on single-tailed, heteroscedastic *t* tests) are indicated above or below a histogram bar to indicate a comparison of that strain to N2 controls, or above a bracket to indicate a comparison between the two strains linked by the bracket. This experiment was performed three times with similar results. **A**, *elo-1* (encoding an FA elongase); **B**, *elo-2* (elongase); **C**, *elo-5* (branched‐chain FA elongase); **D**, *fat-4* (Δ5 desaturase); **E**, *fat‐6* (Δ9 desaturase); **F**, *fat‐7* (Δ9 desaturase).

Transcript steady-state levels were assessed for a panel of genes known to be involved in nematode biosynthesis of lipids. Independent biological samples (*N* = 3 - 8) were prepared from the four longest-lived groups: F1 and F2 homozygotes for the *age-1(mg44)* mutation (median lifespans 2.5- and 10-fold longer than wild-type, respectively); *age-1(hx546)* (1.8-fold); and *daf-2(e1370)* (2.2-fold). First-generation homozygotes for *mg44* receive oocyte contributions from their heterozygous parent (termed “maternal rescue”), blunting the more extreme traits associated with their F2 progeny. Additional groups tested include N2DRM adults (wild-type, lifespan defined as 1.0), N2DRM dauer larvae (developmentally arrested at an alternative larval stage 3), and double mutants of each *age-1* allele with *daf-16* (not shown), which largely or entirely revert longevity and other traits of those mutations [7,36]. Shifts in transcript levels, such as those shown in Figure 3, were highly reproducible; similar results were obtained in three replicate experiments, for all changes that were either significant or suggestive (*P*<0.1) in individual experiments.

The longest-lived group, *age-1(mg44)* F2 adults, showed downregulation for the elongase genes *elo-1*, *elo-2*, and *elo-5*, by ~4-fold [*P*<10^−5^], 2.5-fold [*P*<10^−2^], and 13-fold [*P*<10^−4^], respectively, each relative to N2DRM controls (Figure 3, A-C). However, far smaller decreases were seen in first generation (F1) *age-1(mg44)* homozygotes. A fourth elongase gene, *elo-6*, did not alter significantly in any strain (not shown). Transcript levels of *elo-1*,*-2* and *-5* were also low in N2 dauer larvae. Neither *daf-2(e1370)* nor *age-1(hx546)* adults showed significant decreases for any of the *elo* genes, and *daf-2* mutants instead exhibited a modest (1.7-fold) increase in *elo-2* expression. Thus, transcriptional attenuation of elongase expression appears to be a mechanism of reducing lipid chain length (ACL, Figure 2C) peculiar to *age-1(mg44)* mutants and dauer larvae.

Transcript levels were also assessed for seven nematode fatty-acid desaturase genes. Expression of the *fat-1*, *fat-2* and *fat-3* genes (respectively encoding ω-3, ∆12 and ∆6 desaturases), did not alter significantly in any of the strains examined (data not shown). Transcripts of *fat-4*, encoding a Δ5 desaturase that converts C20:3Δ8,11,14 (n-6) to arachidonic acid, C20:4Δ5,8,11,14(n-6), declined only in F2 *age-1(mg44)* worms, by 2- to 7-fold in three experiments (e.g., Figure 3 D), consistent with the decline in PUFAs observed in longer-lived strains (e.g. Figure 2B). In contrast, two genes encoding Δ9 desaturases, *fat-6* and *fat-7*, both increased similarly in first- and second-generation *age-1(mg44)* homozygotes: two-fold for *fat-6* and 1.6- to 1.8-fold for *fat-7* (Figure 3 E,F). Lesser shifts in the same direction were also seen for *fat-6* in *age-1(hx546)*and *daf-2(e1370)* mutants, and for *fat-7* only in *daf-2(e1370)* adults. In marked contrast to our observations for elongase genes, the desaturase gene shifts observed in *age-1(mg44)* F2 adults either did not occur in dauer larvae (D, *fat-4*), or levels shifted in the opposite direction (E, *fat-6*, and F, *fat-7*). These expression changes seen in longer-lived strains are presumed to underlie their striking increases in Δ9 MUFAs (Table 2; Figure 2, B and H).

### Functional consequences of altering lipid-biosynthesis gene expression

**Figure 4.**
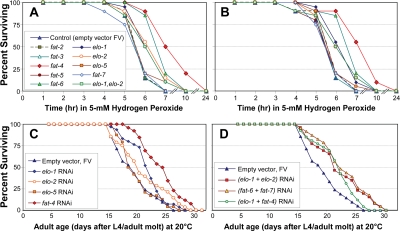
Survival of peroxide stress, and unstressed longevity, of *C. elegans* adults after elongase- or desaturase-specific knock-down by RNAi. **A, B**: Worms (25 per group) were fed on *E. coli* expressing either the indicated siRNA construct (Ahringer RNAi library [39]) or empty-vector control, for 3 days beginning at the L4/adult molt. They were then placed in bacteria-free medium containing 5-mM hydrogen peroxide, freshly diluted from stock solution, and survival was monitored as described previously [7,37,38]. **A** and **B** show replicate experiments, assessing effects of RNAi's targeting desaturase (*fat-2, -3, -4, -5, -6* or *-7*) or elongase genes (*elo-1, -2* or -*5*). **C, D**: Worms (35 worms per group, on 2 plates) were fed the same RNAi constructs as above, continuously from the L4/adult molt, and their survival monitored as described [7,37,38]. **C**, strains treated with single RNAi constructs as indicated; **D**, strains treated with pairs of RNAi constructs, following a protocol for combining RNAi treatments [71]. **C** and **D** together comprise a single experiment, with which replicates (not shown) agreed.

Because all three elongase genes were expressed at reduced levels in the longest-lived (IIS-mutant) worms, while desaturase gene transcript levels varied less systematically, we tested the functional importance of such shifts in expression, for nematode survival of oxidative stress, a rather robust surrogate for longevity effects of IIS disruption [7,37,38]. Wild-type (N2DRM) worms were fed for 3 days, starting at the L4/adult molt, on bacteria expressing gene-specific siRNA constructs [39], and were then assessed for their duration of survival in medium containing a toxic level of hydrogen peroxide. Typical results from two independent experiments are shown in Figure 4 A & B.

Knock-down of *elo-1* or *elo-2* consistently and significantly extended survival of this oxidative stress (each *P*<0.01), whereas RNAi against*elo-5* (encoding an elongase specific for monomethyl branched-chain fatty acids) was no more effective than empty vector (Figure 4). Knock-down of *fat-4*, encoding a Δ5 desaturase, increased peroxide survival time by at least 60% in multiple experiments (each *P*<10^−6^), while depletion of *fat-6* transcripts extended survival by 24% (each *P*<10^−5^). With the exception of the latter result, the effects of RNAi were entirely consistent with expectations based on RNA transcript abundance shifts in the longest-lived strain, *age-1(mg44)*.

Effects of RNAi knockdown on longevity (Figure 4, C & D), largely paralleling conferral of peroxide resistance, agreed even more closely with predictions based on transcript changes in *age-1(mg44)* (see Table 4). Knockdown of either *elo-1* or *elo-2* elicited moderate and significant extensions of *C. elegans* lifespan (each *P*<0.04). RNAi to *elo-5* is not expected to affect longevity, since ELO-5 extends only monomethyl branched-chain fatty acids involved in developmental signaling [27,28]. Combined knock-down of both elongases (Figure 4D) was more effective than depletion of either elongase gene alone, but less than their sum; this could reflect the known redundancy between their functions [40], or simply the dilution of each bacterial strain. The greatest life extension (25%, *P*<10^−4^) followed depletion of *fat-4* transcripts, which also produced the greatest peroxide resistance (60%). In contrast, knockdown of *fat-6* or *fat-7*, both upregulated in long-lived mutants, effected small and insignificant reductions in longevity (Table 4). Life-span changes presented here agree remarkably well with previous data on survival of worms exposed to RNAi's targeting *elo-2, fat-3, fat-4, fat-6* and *fat-7* [10,12].

**Table 4.** Fatty-acid biosynthetic activities that covary with longevity based on lipid-profiles: Trends with lifespan in inferred activities, transcripts encoding implicated enzymes, and effects of RNAi knock-down

Enzyme Activity (*genes* responsible)	Correlation to Lifepan Based on Lipid Profiles	Transcript Change in Long-Lived Mutants	Effect of RNAi KD on Peroxide Resistance of N2DRM Adults (*P by exp.*)	Effect of RNAi KD on Longevity of N2 Adults (*P from log-rank tests*)
Elongases (*elo-1, -2, -5*)	ACL, *R*= −0.87 (*P*<0.002) Elongation ratios: *R*= −0.86 à −0.94 (each *P*<0.002)	*elo-1,* ↓ 3.8x *elo-2,* ↓ 2.6x *elo-5,* ↓ 13x (MMBC ELO)	*elo-1*: ↑14% (0.01, 0.01) *elo-2:* ↑14% (0.01, 0.01) *elo-5:* NC (NS) *elo-1,elo-2*: ↑12% (0.04, 0.03)	*elo-1*: ↑11% (0.02) *elo-2*: ↑8% (0.04) elo-5: NC (NS) *elo-1 + elo-2*: ↑15% (0.004)
Δ6 desaturase (*fat-3*)	*R*= −0.75 (*P*<0.02)	NC	*fat-3,* NC (NS)	*fat-3*: ↑≥10% [10,12] (not retested here due to unaltered H_2_O_2_-survival)
Δ5 desaturase (*fat-4*)	*R*=−0.71 (*P*<0.03)	↓ 2 - 7x in *age-1*(*mg44*) ↑ 1.4-2x in *age-1*(*hx546*) ↑ 1.5-3x in *daf-2(e1370*)	*fat-4*, ↑60% (1E-6, 1E-6)	*fat-4*, ↑ 25% (4E-5) *elo-1* + *fat-4*: ↑12% (differs from FV or *fat-4*, *P*≈0.01)
Δ9 desaturases (*fat-5, -6, -7*)	*R*= +0.94 (*P*<5E-5) for 3 Δ9 desaturases combined**	*fat-5*, NC *fat-6* ↑ 2.0-2.2x *fat-7* ↑ 1.5-1.8x	*fat-5*: NC (NS) *fat-6*: ↑24% (1E-5, 1E-5) *fat-7:* NC (NS)	*fat-5*: NC (NS) *fat-6*: ↓9% (NS) *fat-7*: ↓8% (NS)

**NC:** no change; **NS**, not significant. Life extension by fat-4 RNAi was also reported previously as ~25% [10]. Significance of survival differences was assessed by Cox-Mantel log-rank test, relative to Feeding Vector (FV) control plasmid unless otherwise indicated.

**Figure 5.**
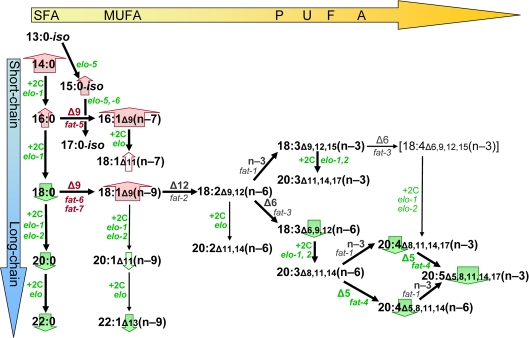
Lipid biosynthesis changes associated with *C. elegans* longevity. Black arrows in this schematic diagram indicate successive steps in fatty-acid biosynthesis by *C. elegans*, based on previously published data [24,25]. Enzyme activities (“+2C” for elongases, “Δn”, “n-3” or “n-6” for desaturases) and the implicated genes are indicated beside each arrow, in green font to indicate that transcripts encoding that enzyme are downregulated with increasing life span, or in red to show upregulation. Block arrows behind the names of fatty-acid classes are similarly color-coded, with the width of the arrow corresponding to the strength of the correlation to longevity. Lipids increase in melting temperature with increasing chain length and/or saturation level; thus shifts toward shorter chains with less desaturation, as seen in long-lived strains, may be neutral with respect to membrane fluidity.

Hypomorphic mutations to genes implicated in the present studies have been demonstrated previously to alter lipid profiles in the directions we found to be associated with lower transcript levels [24-26,41]. For example, mutation to *elo-1* (which shows substantial functional redundancy with *elo-2* [40]) has markedly reduced long-chain FAs and hence PUFAs [26]. A mutation in *fat-4* (encoding the Δ5 desaturase) effectively eliminates specific PUFAs such as 20:5(n-3) and 20:4(n-6) [26]; whereas individual mutations in *fat-5, -6* or*-7* (encoding Δ9 desaturases) lead to significant reductions in either 16:1 or 18:1 Δ9 fatty acids [41]. A schematic of lipid biosynthetic pathways (Figure 5), illustrating alterations to FA abundance and transcript levels in longevity mutants, is thus congruent with the corresponding diagrams based on data from lipogenesis mutants [24-26,41].

**Figure 6.**
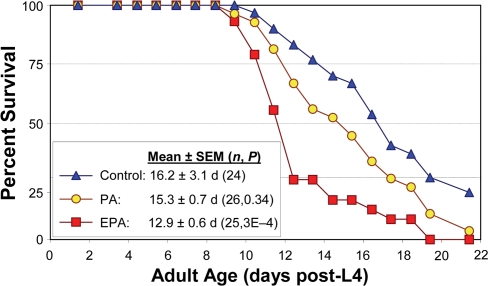
Reduction in *C. elegans* lifespan with addition of PUFAs to their diet. Adult worms were maintained on agar plates spotted with *E. coli* (strain OP50). Control plates were unsupplemented, while treatment plates contained either palmitic acid (PA, 16:0) or eicosapentaenoic acid (EPA, 20:5 (ω-3)). Worms were transferred daily to fresh plates of the same type. Survival in the presence of 40 μM EPA was reduced 20% relative to unaugmented controls (*P*<0.0005 by Gehans Wilcoxon log-rank test), and by 16% relative to worms supplemented with PA approximately isocaloric to the added EPA (*P*<0.007). Each group initially comprised 35 worms, with 24-26 “natural deaths” after censoring worms killed by other factors (see [7,34]).

Another way to perturb the fatty-acid composition of worms is through diet. We fed worms on the standard monoxenic bacterial diet, either unmodified or supplemented with specific fatty acids. As illustrated in Figure 6, the longevity of wild-type Bristol-N2 [DRM] adult worms was significantly reduced (*P*<0.01) by addition of 40 μM eicosapentaenoic acid [EPA, C20:5(ω-3)], the longest-chain PUFA we observed in this nematode, relative to worms given an isocaloric supplement of palmitic acid (C16:0), an unsaturated, shorter-chain fatty acid. Lifespan was also reduced relative to worms not given any supplement. These results are entirely consistent with the trends observed among strains, and the life extensions produced by interference with expression of genes involved in PUFA synthesis.

## DISCUSSION

Comparing lipid composition for a panel of ten nematode strains, 19 of 21 fatty-acid measures showed highly significant (*P*<0.002) linear correlations to log_[10]_ of lifespan, and 17 had at least nominally significant (*P*<0.05) rank-order correlations. We used the logarithm of lifespan for consistency with prior studies (e.g., [34,35]), but there is no a priori reason why the mathematical relation should be log-linear, and overall the data had better fit to a linear plot about as often as they favored log-linear (see Figure 2). The best correlates of lifespan produced Pearson correlation coefficients ranging from −0.94 to +0.96, treating each strain as a single point, or −0.80 to +0.93 taking each independent biological preparation as one point (Table 2 and Supplemental Table S3). The “fraction of variance explained” by a correlation is *R*^2^, from which the ten parameters with highest *R* values would individually account for 45-92% of the total variance in longevity. Obviously, there were enough strong predictors of longevity to account for all the variance among strains several times over ― a clear indication that these parameters are themselves inter-related.

Might all of these longevity-associated FA traits simply reflect their common derivation from increased storage lipids, triacylglycerides (TAG)? TAGs were previously noted to be elevated in certain *daf-2* mutants, although not correlated with the lifespans of those alleles [9]. Based on Sudan black staining (data not shown), storage lipid vesicles are indeed elevated more (~2x N2) in very long-lived *age-1(mg44)* F2 adults, than in adults of the less long-lived mutants *age-1(hx546)* and *daf-2(e1370)* (1.4-1.7x). At first glance, this unitary explanation appears attractive because TAGs of wild-type N2 worms are enriched for the shortest-chain FAs (C14:0 and C15-*iso*) and some MUFAs, but have less C18:0 and PUFAs than are present in the phospholipid fraction [24]. However, the changes we report here in individual FA chains are quite inconsistent with that interpretation. For *daf-2(e1370)*, 8 of the 19 FA chains assessed actually differ in the opposite direction from that reported for TAGs, while two that vary in the same direction would require a 10-fold or greater increase in the TAG fraction, relative to N2, to account for our observations. In the case of *age-1(mg44)*, 6 FAs moved in the opposite direction to TAGs, and 8 FAs would require TAG increases of 10- to 30-fold.

We here propose a different “reductionist” explanation that is consistent with our data. Nearly all of the FA trends associated with lifespan could be attributed to two main factors: reduced elongase activities, and increases or declines in specific desaturase activities, in the longest-lived IIS mutant strains. These inferences were borne out by direct analysis of gene expression levels for many of the implicated enzymes. In the longest-lived strains, transcript levels were reduced for three elongases and a Δ5 desaturase (which contribute to PUFA formation), but increased for both Δ9 desaturases (which form oxidation-resistant MUFAs).

Functional assays, using RNAi to attenuate gene expression, provide evidence that such genes might play causal roles in enhancing lifespan. Longevity extensions permit stronger inferences than reductions, due to the many ways lifespan could be shortened upon disruption of any pathway that contributes to survival. It is particularly intriguing that survival of wild-type *C. elegans*, under either peroxide stress or benign conditions, benefits from RNAi suppression of genes encoding either of two elongases or a Δ5 desaturase, *fat-4*, whereas knockdowns of Δ9-desaturase genes can slightly reduce longevity.

Taken together, these functional data imply that modulation of FA composition to increase resistance to lipid peroxidation is one of the mechanisms by which IIS mutants extend longevity. This is supported by recent imaging studies of coherent anti-Stokes Raman scattering (CARS) and Raman spectral analysis of lipid unsaturation, in which three IIS-related mutants (*daf-2(e1370)*, *rict-1(ft7)* and *sgk-1(ok538)*) registered 35 - 40% reductions in lipid unsaturation, although only *daf-2* had increased fat stores (by ~2-fold) relative to N2 wild-type worms [42].

Only one of our results was inconsistent with the above interpretation: oxidative-stress survival was enhanced by RNAi to *fat-6*, encoding a ∆9 desaturase, whereas lifespan was modestly decreased (as expected) by knockdown of either *fat-6* or *fat-7*. It is noteworthy that combined RNAi targeting both *elo-1* and *fat-4* (Figure 4D) produced almost precisely the same survival profile as knockdown of *elo-1* alone, losing the greater benefit of *fat-4* knockdown (Figure 4C). Epistasis offers a plausible explanation for that outcome, in which absence of *elo-1* impairs the synthesis of longer-chain fatty acids, which are therefore not available for conversion to PUFAs via the FAT-4 desaturase.

A point-by-point accounting for our results is as follows. ELO-1 and ELO-2 are required for fatty acid elongation beyond C14:0 (Figure 5), and so knock-down of their expression (especially that of *elo-1*, which extends shorter-chain FAs) may be sufficient to block or reduce PUFA formation. In contrast, *elo-5* and *elo-6* are specialized to elongate monomethyl branched-chain FAs, producing C17:0-*iso* [27,28], a developmental signal that nutrition is adequate for larval progression [28]; their downregulation in adults is thus not expected to alter longevity. The ∆9 desaturases create MUFAs, poor substrates for lipid peroxidation which are thus thought to favor longevity [43]. ∆9 MUFAs increase with strain lifespan, as does expression for two of the three ∆9 desaturase genes (*fat-6* and *fat-7*, but not *fat-5*), while knock-down of *fat-6* or *fat-7* decreases lifespan of normal adults.

PUFA formation requires additional desaturases, in particular those encoded by *fat-3* and *fat-4*. RNAi targeted to *fat-3*, reducing expression of the nematode ∆6 desaturase, was reported previously to benefit longevity by 10-15% [10,12], although no significant changes were seen for transcript levels in long-lived mutants, or for peroxide survival in wild-type worms exposed to *fat-3* RNAi. FAT-4, the nematode ∆5 desaturase, is involved in formation of the most desaturated (and thus most peroxidizable) fatty acids, which might account for the disproportionate benefit to peroxide resistance and longevity conferred by *fat-4* knockdown (Figure 4 and Table 4).

Fatty acid quantitation data are summarized graphically in Figure 5, which has been redrawn from biosynthetic pathways established previously [25,26]; changes in gene expression for key enzymes are indicated by font color. Considered in the context of functional tests with RNAi knockdown, these data strongly implicate shifts in the fatty acid profile toward decreased poly-unsaturation and shorter chain lengths, as contributing factors to strain longevity increases conferred by IIS-disrupting genetic mutations. Such changes could be orchestrated by regulatory genes such as *nhr-49* [25,44] or *nhr-64* [45], encoding nuclear hormone receptors with roles in the coordination of lipogenesis (paralleling mammalian PPARs [44]) and lipid oxidation [45], respectively.

Although the data implicating these changes in fatty-acid chain structure appear to be primarily relevant to insulinlike signaling, the RNA interference data indicate survival roles for the corresponding lipid-biosynthesis genes in wild-type worms. The paramount influence of IIS genes, in defining structural trends across the mutant panel, reflects the fact that the three longest-lived mutations disrupt IIS. This is a difficult situation to avoid, given that no other mutations produce lifespan effects of comparable magnitude to IIS impairment. Several mutations reported to elicit life extensions approaching those of IIS proved considerably less effective after outcrossing into the N2DRM background.

The data reported here provide important new insights into longevity regulation. They imply that fatty-acid composition, and thus resistance to lipoperoxidation, constitutes one of the causal mechanisms contributing to normal lifespan, and through which IIS mutations confer life-extension. In this view, the decline in peroxidation index (PI) with increasing lifespan would impart survival benefits by reducing the principal substrates of lipoperoxidation, a process responsible for the only free-radical chain reactions of biological significance [32]. Lowering PI, however, also reduces membrane fluidity - which in poikilotherms must ultimately limit the extent to which PI can be reduced. Altering fatty-acid saturation is a well-documented mechanism by which free-living nematodes adjust to temperature fluctuations encountered in their natural environment, thereby stabilizing membrane fluidity [46,47]. The present report is the first, to our knowledge, to indicate that shifts in fatty-acid chain length may play a similar role.

These observations have important implications for human health, which depends in diverse ways on dietary lipid intake, and for human longevity, which is profoundly impacted by both obesity [48-50] and insulinlike signaling [51-53]. A recent examination of the lipid composition of erythrocyte membranes in the offspring of nonagenarians, compared to age-matched controls (whose parents were of less exceptional longevity) indicated shifts in lipid composition that are remarkably similar to those reported here [54]. Specifically, adult progeny of nonagenarians had significantly elevated levels of MUFAs, in particular C16:1Δ9 and *trans* C18:1Δ9, but reduced total PUFAs, and especially C20:4, compared to age-matched controls [54]. Of particular interest were significant reductions among nonagenarian progeny, of the peroxidation index (PI) by 25%, and of the unsaturation index (UI, equivalent to the DBI) by 19% [54] ― parameters that declined by 44 and 21%, respectively, in the longest-lived worms relative to N2 controls. The shorter-chain saturated fatty acids (C16:0, C18:0) also increased in nonagenarian offspring, although this fell short of statistical significance. Thus, extreme longevity in humans, as in worms, features marked reductions in lipid peroxidation substrates, offset by large increases in the shorter MUFAs (C16:1, C18:1) and lesser gains in short-chain saturated fatty acids.

The one noteworthy difference between these otherwise parallel outcomes was that essentially all of the decrease in PUFAs among nonagenarian progeny occurred in the ω-6 (n-6) PUFA class, whereas in nematodes both ω-6 and ω-3 PUFAs fell with increasing lifespan (Tables 2 and S3a). This difference likely reflects the vast excess of (n-6) over (n-3) fatty acids in typical western diets [55], with ratios ranging from 10 to 30, in marked contrast to nematodes wherein the balance of these competing PUFAs is largely determined by endogenous lipid metabolism.

An inverse correlation between peroxidation index and lifespans of mammalian species has been noted previously [56]. Examples in which extreme longevity is accompanied by membranes of exceptionally low peroxidizability include the naked mole rat [34], the monotreme mammal echidna [57], and, as already mentioned, humans [54]. A similar correlation was also reported within an invertebrate species: the long-lived honeybee queen has PUFA levels well below those of short-lived workers [58]. These comparative studies cannot, by their nature, permit an unambiguous distinction between cause and effect. For example, low susceptibility to lipid peroxidation might favor longevity, or might have evolved secondarily in longer-lived species (or castes in social animals) which require their cell membranes to endure longer. The ability to experimentally manipulate transcript levels in *C. elegans* enabled us to assess the consequences of reducing the expression of genes encoding fatty acid desaturases or elongases. Although the effects observed were relatively modest, they were in the direction predicted if reduced FA chain length and desaturation both favor greater longevity. In conjunction with our previous functional-intervention studies, which indicated that increasing or decreasing enzymatic defenses against lipoperoxidation had a corresponding effect on nematode longevity [32,33], the present data support the hypothesis that the potential for fatty-acid oxidization is a key factor through which IIS mutations (and possibly others) modulate nematode lifespan.

## MATERIALS AND METHODS

### Caenorhabditis elegans strains

Reputedly long-lived strains examined in this study were CB928, DA820, TJ3014, CB1370, DR26, MQ514, and GR1168 (provided by the Caenorhabditis Genetics Center [CGC], Minneapolis MN). These strains were made congenic (near-isogenic) by six consecutive out-crossings of each against a single male-enriched stock of Bristol-N2 wild-type worms (CGC “N2 male” stock; the Riddle-laboratory designation is N2DRM). The *daf-16(m26)* mutation largely or completely reverses life extension by *age-1(mg44)* [7], similar to its reversion of traits arising from *daf-2* and other *age-1* mutations [5,6,59]. Double mutants combining *age-1* or *daf-2* with *daf-16(m26)* were created by crossing several of the above outcrossed strains to DR26 [*daf-16(m26)*], similarly outcrossed, and identified among progeny of those crosses by genotyping via polymerase chain reaction (PCR) after propagation. The *age-1(mg44)*mutation is maintained as a mixed heterozygous stock in its original strain (GR1168) and in our N2DRM-outcrossed strain (SR808), with a wild-type *age-1* allele provided on the *mnC1* balancer chromosome to suppress homologous recombination [60]. From the SR808 stock, *age-1* homozygotes were segregated as needed, and their “F2” adult progeny (which are slow-developing and infertile) were harvested after attaining approximately normal adult size (day 8 - 11 after the L4/adult molt). The congenic strains constructed, and their relative longevities, are summarized in Table 1.

### Nematode maintenance and harvesting

Worms were cultured at 20°C on “NG” plates (supplemented with peptone to 2% w/v) seeded with lawns of *E. coli* var. OP50 [61]. At harvest, worms were held without food for 30 min at 20° C, which we found sufficient for digestion of gut bacteria and degradation of bacterial DNA to below detectable limits [62]. Worms were harvested as young adults (unless noted otherwise) to avoid confounding of lifespan effects with senescence effects. Worm pellets were promptly frozen on dry ice and stored at −80°C. Replicate assays of *C. elegans* strains used independent worm expansions and cultures to assess inter-cohort variance, which could also contribute to inter-strain variation.

### Determination of lifespan

Nematodes, grown as described above, were rinsed from plates with S buffer (0.1 M NaCl, 0.05 M potassium phosphate, pH 6.0) [63-65]. Adults, enriched and recovered by settling, were resuspended in alkaline hypochlorite (0.5-N NaOH, 1.05% hypochlorite) and lysed during 5 min incubation at 20° C. The recovered eggs were rinsed in S buffer and transferred to fresh agar plates seeded with *E. coli* (strain OP50). Survival cultures were begun on 60-mm agar plates, one day after the L4/adult molt, transferring 50 adults (25 per plate) to 60-mm dishes containing NGM agar and a central lawn of OP50 *E. coli*. Cultures were maintained at 20°C and live worms counted daily upon transfer to fresh dishes. Worms that failed to move, either spontaneously or in response to touch, were scored as dead. Worms lost (stranded on Petri-dish walls or within the agar), or killed by internal hatching of progeny, were censored at the midpoint of the time span between observations, whereas worms inadvertently killed were censored at the time of the event. Adult lifespans of *C. elegans* strains (Table 1) are calculated as mean or median days of survival after the L4-adult molt, normalized to the lifespan of the N2DRM strain, identically assessed as a simultaneous control. This departs from the more common practice of expressing lifespan as days of survival after eclosion of larvae from eggs, but avoids confounding the effects of mutations on developmental timing and longevity [66].

### Reagents

Chemicals used in GC sample preparation (1-docosanol, chloroform, pyridine, methanol, and hydrochloric acid) were purchased from Regis Technologies Inc. (Morton Grove, IL). Chemicals for buffers, and a Supelco 37-FAME set of GC-MS standards, were obtained from Sigma-Aldrich (St. Louis, MO).

### GC-MS sample preparation

Frozen pellets were pulverized to fine powders under a dry nitrogen gas atmosphere in mortars chilled on dry ice. Frozen powder samples were lyophilized at −60° C under reduced pressure (<100 mTor) for 27 h followed by 1 h at −20° C and then 30 min at 15° C. Lyophilized samples weighing 4.00-4.05 mg were soaked in 1 ml of chloroform overnight at −80° C. A solution of 1-docosanol in 15 μL of chloroform was added at 15 μg per sample as an internal standard to monitor the efficiency of recovery through all processing and analytic steps. Samples were brought to 0° C for 1 h and sonicated repeatedly in an ice-water bath for a total of 10 min. Two successive liquid-liquid extractions were performed after additions of de-gassed, phosphate-buffered water (0.5-mM, pH 7.4). The organic phases were dried under a nitrogen stream at room temperature, whereas aqueous phases (not analyzed here) were deproteinized, dried and stored. Organic extract residues were dissolved in a solution of 1.25-M HCl in methanol. Incubation for 4 h at 50° C resulted in methyl-transesterification of esters, thus generating fatty acid methyl esters (FAMEs) from lipids. After desolvation, the residues were reacted with at least a 2:1 excess of the trimethylsilyl donor MSTFA (including 1% TMCS as catalyst; Pierce, Rockford IL), dissolved in pyridine. After incubation for 1 h at 50° C, reactions were chilled and subjected to GC-MS analysis within 24 h.

### GC-MS analysis

GC-MS was performed on an Agilent 5890 II+ gas chromatograph coupled to an Agilent 5972 mass spectrometer. Each sample was introduced into a DB-5MS capillary column (J&W Scientific), 60-m long × 0.25-mm ID with 0.25-μm film thickness, integrated with a 5-m guard column. Automatic injections of 0.5-μl samples were made, without splitting, into the GC inlet set to 280°C. The thermal program began at 80° C for 2 minutes, then increased linearly to 210° C at a ramping rate of 6° C/min, further increased to 310° C at the slower ramping rate of 2.5°C/min, and held this temperature for 8 min. Helium was used as the carrier gas with a constant flow rate of 1 ml/min under electronic pressure control. Positive 70-mV electron-impact quadrupole ion scans were acquired every 0.75 sec, from 650 to 50 m/z. Each run was initiated with an 11.5-minute solvent delay before ionization commenced, to bypass the pyridine peak. The transfer-line temperature was set to 280°C and the indicated detector temperature was 172° C. Detection parameters were tuned using the maximum-sensitivity autotune mode. A blank sample (pyridine only), a blank supplemented with four long-chain alkane retention-index (RI) standards, and a sample containing the same RI standards and a derivatized recovery standard used in quantitation, were run at the beginning, the middle and the end of each sequence of runs, for quality-control purposes.

### Quantitation of transcript abundances by real-time polymerase chain reaction

Gene expression was assessed by real-time quantitative polymerase chain reaction (RT-qPCR) to amplify cDNAs reverse-transcribed from total RNA, as described [37]. Total RNA was purified (RNeasy, Qiagen) from each group, copied to DNA by reverse transcriptase (SuperScript III, Invitrogen), and the products quantified by RT-qPCR on an Opticon2 thermal cycler (MJ Research), using SYBR Green stain (Roche).

### Data analysis and statistics

The first step in quantitation of fatty acids is the alignment of peaks across all GC-MS runs, utilizing both chromatographic information (retention times) and mass-spectral data (m/z components) to establish the chemical identity of peaks being compared. To correct retention time variation between runs, we applied an iterative block-shift alignment method [67] to produce precisely aligned data with minimal peak distortion (thus preserving quantitative information), resulting in 77 three-way arrays (m/z × time × samples). Each array was independently decomposed into three matrices (relative concentrations, elution profile, and mass spectrum), by PARAFAC analysis [68] using a MATLAB script (Version 3.10) from the N-way Toolbox [69]. Compounds corresponding to the deconvoluted mass spectra were identified by matching to the NIST-08 Mass Spectral Library, with minimal criteria of >80% for both AMDIS (Version 2.66) peak purity and the NIST matching index. All compounds identified as fatty acid methyl esters (FAMEs) agreed closely, in retention times and spectra, to corresponding analytes in a reference set comprising 37 FAMEs.

Fatty acid quantities obtained by GC-MS were normalized in two ways: (1.) to an internal standard (1-docosanol, 15 μg added to each sample prior to sonication and lipid extraction), or (2.) to the sum of all lipid peaks identified. Expression of peak areas as ratios to the area of the internal standard peak, observed on the same chromatogram, adjusts for run-to-run variation in the efficiencies of extraction, reaction with derivatizing agents, and detection. At the same time, this normalization preserves strain-to-strain differences in the absolute amounts of individual or summed FA components, since dry mass was accurately measured for lyophilized samples, and loads were thus standardized prior to GC-MS analysis. In the second normalization procedure, relative FA amounts were obtained by dividing each peak area by the sum of areas for all FA peaks appearing on the same chromatogram, which thus serves as an unbiased, endogenous internal standard (Table 2).

Associations between lifespan or gravidity and the amounts of FAs were analyzed for data organized by strain, using parametric and nonparametric analysis of correlation (Table 2) or one-way ANOVA (Table 3). In pairwise post-hoc comparisons, Dunnett's procedure was used to compare other strains to N2DRM as the normal-lifespan reference group. For correlation analysis of trends in young-adult worms as functions of lifespan, strains were grouped into bins if their longevities could not be unambiguously distinguished. Lifespans relative to wild-type (N2DRM) are summarized in Tables 1 and S1.

Correlations between the various lipid parameters and longevity were evaluated in two ways: (1.) as Pearson correlation coefficients (*R*), which describe the linear relationship between parameters; or (2.) as Spearman (Rank-Order) correlation coefficients (*R_S_*), which are nonparametric and hence independent of the distributions of variables and of any assumptions regarding the relationships among variables. In either case, each correlation coefficient *R* was calculated from *N* data points, equal to the number of independent populations harvested and analyzed in separate GC-MS runs. The corresponding *P* value were ascertained by *t*-transformation,*t* = |R| ∙ [(N - 2) / (1 - R2)]^½^, treating the result as coming from a *t* distribution with *N*-2 degrees of freedom [70].

To test for a possible confounding influence of the infertility phenotypes of strain SR808, *age-1(mg44)* (reduced sperm and absence of mature oocytes or retained embryos) on its FA profile, two post-hoc analyses were made. (a.) Three control strains were assessed both as fertile young adults (days 2-3 of adult-hood), and as post-gravid, pre-senescent adults (approximately day 6 of adulthood) with no eggs or mature oocytes observed by microscopic examination; the relative contribution of fertility to FA content, estimated by ANOVA, was not statistically significant, ranging from −33 to +26% of the difference between SR808 the mean of all control samples. (b.) The trend with longevity was reassessed by ANOVA for the subset of nongravid (“eggless”) samples, i.e. strain SR808 and post-gravid (day-6) samples which comprised the above controls plus SR807, *age-1(hx546)*. Equal weight was assigned to each lifespan grouping in this analysis.

In considering the significance of trends in FA levels with longevity, strict Bonferroni adjustment for multiple endpoints shown in Table 2 would require P < 0.05/21, or 0.0024. By this stringent criterion, 13 of the 21 linear correlations would be considered significant, while 7 of the nonparametric correlations qualify. Such a strict threshold is appropriate for measures that are independent of one another, but is overly conservative for lipid biosynthesis wherein successive enzymatic steps of elongation and desaturation produce a series of FA-chain types. The abundance of each FA type depends strongly on the level of its precursors; e.g., C18:0 depends on C16:0 levels, and C18:1(n-9) depends on the level of C18:0, and thus also of C16:0 (see Figure 5). In Table 2, numerous ratios are derived as functions of a subset of 19 measured FA levels, and hence are entirely dependent on those data. Uncorrected *P* values are given here, to allow the reader to choose between strict Bonferroni adjustment, and a less conservative significance threshold such as α = 0.01, commonly applied in similar situations to avoid overinflating type-II errors.

## SUPPLEMENTARY TABLES



## Abbreviations

ACL: average chain length
DBI: double bond index
FA: fatty acid
FAME: fatty acid methyl ester
GC-MS: gas chromatography - mass spectrometry
IIS: insulin/insulinlike growth factor-1 signaling
mmBC: monomethyl branched-chain
MUFA: monounsaturated fatty acid
PI: peroxidation index
PUFA: polyunsaturated fatty acid
RT-PCR: real-time reverse transcriptase PCR
