# Supplementary material for: Modulation of lipid biosynthesis contributes to stress resistance and longevity of C. elegans mutants
Source: Aging (Albany NY). 2011 Feb 25;3(2):125–47. doi: 10.18632/aging.100275 (PMC3082008; doi:10.18632/aging.100275)
Supplement: Supplementary file 1 [file aging-03-125-s001.pdf]

SUPPLEMENTAL TABLES

Supplemental Table S1. Survival (Life Table) Summary for Longevity-Mutant Strains

Strain		Genotype	Median Adult Lifespan (d)	Rel. Median Lifespan	Mean Adult Life-span (d) ±SEM	Rel. Mean Lifespan	N (deaths counted)	C (worms censored)	Diff. from N2, Cox-Mantel log-rank *P*<
N2DRM	a	wild type	**18.0**	**0.97**	**17.9±0.8**	**0.98**	31	4	—
	b		**19.0**	**1.03**	**18.7±0.8**	**1.02**	22	13	
SR803	a	*old-1(zIs3000)*	**19.0**	**1.03**	**19.3±0.7**	**1.05**	33	2	N.S.
	b		**19.0**	**1.03**	**18.9±0.7**	**1.03**	31	4	
SR801	a	*eat-18(ad820)*	**21.0**	**1.14**	**20.5±0.8**	**1.12**	26	7	0.04
	b		**21.0**	**1.14**	**19.6±0.8**	**1.07**	25	10	
SR800	a	*unc-31(e928)*	**21.0**	**1.14**	**24.8±2.3**	**1.36**	14	20	0.001
	b		**23.5**	**1.27**	**24.6±1.4**	**1.34**	22	12	
SR806	a	*daf-2(e1370)*	**41.9**	**2.26**	**37.8±2.0**	**2.07**	29	5	<1E–10
	b		**41.9**	**2.26**	**39.5±1.7**	**2.16**	29	6	
SR807	a	*age-1(hx546)*	**30.1**	**1.63**	**29.9±1.5**	**1.63**	31	4	<1E–10
	b		**30.1**	**1.63**	**30.4±1.2**	**1.66**	33	2	
N2DRM	a	wild type*	**15.5**	**0.95**	**15.1±0.6**	**0.94**	36	6	—
	b		**17.3**	**1.05**	**17.2±0.7**	**1.06**	33	8	
SR808	a	*age-1(mg44)**	**150.4**	**9.2**	**136.4±16.3**	**8.5**	15	33	<1E–15
	b		**161.6**	**9.9**	**170.2±12.0**	**10.5**	14	42	
N2DRM	a	wild type	**17.5**	**0.94**	**17.2±0.9**	**0.99**	27	3	—
	b		**19.6**	**1.06**	**17.5±1.0**	**1.01**	29	1	
SR807	a	*age-1(hx546)*	**33.5**	**1.81**	**27.8±1.8**	**1.60**	21	8	<1E–4
	b		**26.5**	**1.43**	**28.2±1.2**	**1.63**	21	9	
SR819	a	*age-1hx546); daf-16(mu26)*	**19.6**	**1.06**	**17.8±0.8**	**1.03**	28	2	N.S.
	b		**19.6**	**1.06**	**19.3±0.6**	**1.11**	25	5	
SR820	a	*age-1mg44); daf-16(mu26)*	**19.6**	**1.06**	**18.5±0.9**	**1.07**	28	2	0.04
	b		**19.6**	**1.06**	**19.5±0.6**	**1.12**	27	3	

Relative median and relative mean adult lifespans are ratios of each group's value over the mean of N2DRM controls.

Data from duplicate experiments were combined prior to testing of significance. *Data from [7]. N.S., not significant.

Supplemental Table S2. Survival (Life Table) Summary for N2DRM Worms Exposed to RNAi

RNAi Added	Median Adult Lifespan (days at 20˚C)	Mean Adult Lifespan (days) ±SD	Relative Lifespan (Mean/N2)	N (deaths counted)	C (worms censored)	Diff. from N2: Cox-Mantel log-rank *P*<
**Feeding Vector**	**18.7**	**19.6±0.7**	—	21	14	--
elo-1	**22.0**	**21.8±0.6**	**1.11**	27	8	**0.02**
elo-2	**20.1**	**21.3±0.8**	**1.09**	28	7	**0.04**
elo-5	**18.5**	**20.2±0.6**	**1.03**	24	11	**N.S.**
fat-4	**24.6**	**24.5±0.7**	**1.25**	26	8	**4E–5**
elo-1 + elo-2	**21.5**	**22.5±0.9**	**1.15**	26	9	**0.004**
fat-6 + fat-7	**22.4**	**23.2±0.9**	**1.18**	23	12	**0.001**
elo-1 + elo-4	**22.2**	**21.9±0.7**	**1.12**	26	9	**0.006**

Supplemental Table S3a. Fatty acid composition of isogenic *C. elegans* strains of varying lifespan

(N.B.: double-mutant control strains, not shown here, were included to determine correlation coefficients)

	Median Lifespan:	~1X		1		1.14X		1.21X		1.63X		2.3X		9.6X		Pearson		Pearson		Rank-Order	
	Genotype:	*old-1(zls3000)*		Wild Type		*eat-18(ad820)*		*unc-31(e928)*		*age-1(hx546)*		*daf-2(e1370)*		*age-1(mg44)*		Correlation *v.*		Correlation *v.*		Correlation	
	Strain (Biol. *N*):	SR803 (2)		N2DRM (5)		SR801 (2)		SR800 (2)		SR807 (5)		SR806 (3)		SR808 (4)		logLS (*N*=10)		logLS (*N*=36)		*v.* LS (*N* =36)	
		Mean	±SD	Mean	±SD	Mean	±SD	Mean	±SD	Mean	±SD	Mean	±SD	Mean	±SD	*R*	$P_R \leq$	*R*	$P_R \leq$	R_S	$P_R \leq$
mmBC	C15:0 *iso*	3.6	0.0	4.1	0.5	2.4	0.3	3.6	0.8	3.9	0.6	4.4	1.3	4.7	1.3	0.63	0.06	0.41	0.02	0.23	—
mmBC	C17:0 *iso*	5.2	0.02	5.8	0.5	3.7	0.1	4.3	0.2	5.3	0.6	5.1	0.2	5.1	0.8	-0.07	—	-0.18	—	-0.21	—
SATURATED FAs	C14:0	0.9	0.1	1.0	0.2	1.3	0.05	1.1	0.2	1.0	0.2	1.5	0.6	3.0	1.3	0.96	1E-5	0.82	1E-9	0.53	8E-4
SATURATED FAs	C16:0	4.0	0.2	3.8	0.6	4.5	0.4	4.6	1.5	4.0	0.2	4.7	1.0	4.8	0.6	0.67	0.04	0.47	0.004	0.45	0.007
SATURATED FAs	ΣSC-SFAs (C14–C16)	8.4	0.3	8.9	1.0	8.3	0.1	9.3	2.6	8.9	0.8	10.6	2.9	12.7	2.7	0.96	2E-5	0.68	5E-6	0.42	0.01
SATURATED FAs	C18:0	10.47	0.8	9.8	1.9	11.2	0.2	11.6	1.9	10.3	0.7	9.4	2.3	6.5	1.7	-0.75	0.02	-0.57	3E-4	-0.23	—
SATURATED FAs	C20:0	4.0	0.1	3.3	1.0	4.1	0.3	4.7	1.2	3.5	1.7	2.7	0.9	1.5	0.8	-0.81	0.005	-0.52	0.001	-0.40	0.02
SATURATED FAs	C22:0	1.0	0.1	0.9	0.3	1.1	0.2	1.0	0.2	1.0	0.3	0.8	0.1	0.5	0.1	-0.91	3E-4	-0.60	1E-04	-0.43	0.01
SATURATED FAs	ΣLC-SFAs (C18–C22)	15.4	1.0	14.0	2.8	16.4	0.3	17.3	3.3	14.7	2.6	12.9	2.8	8.5	2.4	-0.80	0.006	-0.60	1E-04	-0.34	0.05
MUFAs	C16:1Δ9(n-7)	2.8	0.1	2.7	0.4	2.4	0.1	2.8	0.2	2.2	0.9	4.2	0.9	10.6	1.6	0.95	3E-5	0.93	7E-16	0.53	0.001
MUFAs	C18:1Δ11(n-7)	25.6	0.7	27.7	6.0	21.7	0.7	22.2	1.8	24.3	3.0	28.4	7.7	34.7	3.2	0.71	0.02	0.55	6E-04	0.25	—
MUFAs	C18:1Δ9(n-9)	0.3	0.01	0.7	0.1	0.8	0.2	0.8	0.7	0.6	0.2	0.7	0.5	1.0	0.1	0.52	—	0.29	—	0.19	—
MUFAs	C20:1Δ11(n-9)	1.3	0.1	0.9	0.5	0.9	0.06	0.9	0.4	0.7	0.3	1.0	0.7	0.7	0.2	-0.51	—	-0.27	—	-0.37	0.03
MUFAs	C22:1Δ13(n-9)	1.4	0.9	2.0	0.9	1.8	0.5	2.8	1.1	2.0	0.9	1.4	0.3	1.0	0.5	-0.50	—	-0.37	0.03	-0.15	—
MUFAs	Σ(MUFAS)	31.6	1.9	34.0	5.5	27.7	1.3	29.6	3.6	29.8	3.1	35.8	7.2	48.0	3.8	0.84	0.002	0.74	2E-7	0.28	—
PUFAs	C18:2(n-6)	6.7	0.3	7.6	2.0	7.4	0.8	6.1	1.2	7.1	1.0	6.9	0.2	7.2	0.8	0.08	—	0.05	—	0.09	—
PUFAs	C18:3(n-6)	2.1	0.2	2.1	0.5	2.3	0.2	2.0	0.2	2.1	0.9	1.8	0.3	1.2	0.3	-0.82	0.004	-0.51	0.002	-0.39	0.02
PUFAs	C20:2(n-6)	1.5	0.05	1.4	0.6	1.7	0.3	1.4	0.7	1.0	0.3	1.3	0.5	1.6	0.2	-0.01	—	0.05	—	-0.06	—
PUFAs	C20:3(n-6)	4.4	0.2	3.6	0.6	4.9	0.1	4.5	0.7	4.3	0.3	4.8	1.3	3.5	0.7	-0.24	—	-0.18	—	0.03	—
PUFAs	C20:4(n-6)	4.4	0.06	3.9	0.9	4.1	0.2	4.2	0.8	4.2	0.7	3.4	0.7	2.7	0.8	-0.89	5E-4	-0.55	6E-04	-0.46	0.005
PUFAs	C20:4(n-3)	5.3	0.07	4.5	1.0	6.9	0.1	5.9	0.4	5.3	0.3	5.1	1.4	3.2	0.9	-0.64	0.05	-0.54	8E-04	-0.26	—
PUFAs	C20:5(n-3)	14.9	0.04	14.3	2.4	16.5	0.1	15.4	1.4	17.3	1.3	12.3	1.0	6.4	0.9	-0.85	0.002	-0.80	6E-09	-0.43	0.01
PUFAs	Σ(PUFAS) ≥3 desat sites	31.2	0.3	28.4	4.8	34.9	0.4	32.1	0.1	33.2	1.2	27.4	2.9	17.0	3.2	-0.82	0.004	-0.77	6E-08	-0.36	0.03

Supplemental Table S3b. Fatty-acid ratios implying lipid-synthetic activities

(N.B.: double-mutant control strains, not shown here, were included to determine correlation coefficients)

Median Adult Lifespan:	~1X		1		1.14X		1.21X		1.63X		2.3X		9.6X		Pearson Correlation to log LS (N =10)		Pearson Correlation to log LS (N =36)		Rank-Order Correlation to LS (N =36)	
Genotype:	*old-1(zIs3000)*		WT (N2DRM)		*eat-18(ad820)*		*unc-31(e928)*		*age-1(hx546)*		*daf-2(e1370)*		*age-1(mg44)*							
Strain(Biol. *N*):	SR803 (2)		N2DRM (5)		SR801 (2)		SR800 (2)		SR807 (5)		SR806 (3)		SR808 (4)							
	Mean	±SD	Mean	±SD	Mean	±SD	Mean	±SD	Mean	±SD	Mean	±SD	Mean	±SD	R	$P_R \leq$	R	$P_R \leq$	R_S	$P_R \leq$
ACL	18.2	0.2	18.0	0.2	18.2	0.1	17.9	0.1	18.1	0.2	18.0	0.1	17.6	0.2	-0.87	0.002	-0.63	5E-5	-0.49	0.003
DBI	181.4	0.6	174.2	12.0	194.6	2.6	181.6	10.2	189.5	6.9	167.5	7.4	135.2	9.0	-0.84	0.003	-0.78	2E-8	-0.40	0.02
PI	150.8	0.4	140.8	19.8	167.8	3.0	154.2	7.0	163.3	7.6	130.2	9.7	81.5	11.9	-0.85	0.002	-0.80	7E-9	-0.39	0.02
ω3/ω6 ratio	1.06	0.04	1.03	0.24	1.15	0.06	1.18	0.09	1.21	0.08	0.96	0.11	0.60	0.08	-0.81	0.005	-0.70	2E-6	-0.37	0.03
**Δ9 Desaturase Activity**																				
C16:1Δ9/16:0	0.72	0.08	0.71	0.13	0.54	0.02	0.63	0.16	0.54	0.25	0.88	0 .02	2.23	0.29	0.93	1E-4	0.90	5E-14	0.35	0.04
C18:1Δ9/18:0	0.028	0.003	2.98	1.06	1.93	0.02	1.96	0.48	2.37	0.40	3.30	1.81	5.60	1.58	0.79	0.007	0.61	8E-5	0.28	—
Total Δ9 activity	0.22	0.03	0.25	0.04	0.20	0.01	0.23	0.08	0.19	0.06	0.34	0.03	1.05	0.24	0.94	5E-5	0.91	4E-14	0.35	0.04
**Δ5 Desaturase Activity**																				
C20:4(n-6)/ C20:3(n-6)	1.00	0.07	1.10	0.14	0.83	0.01	0.92	0.03	0.99	0.22	0.75	0.27	0.75	0.09	-0.71	0.03	-0.44	0.008	-0.56	5E-4
**Δ6 Desaturase Activity**																				
C18:3(n-6)/ C18:2(n-6)	0.31	0.02	0.29	0.11	0.32	0.06	0.33	0.10	0.30	0.11	0.26	0.04	0.17	0.07	-0.75	0.02	-0.50	0.003	-0.37	0.03
**Omega-3 Desaturase Activities**																				
C20:5(n-3)/ C20:4(n-6)	3.37	0.04	3.70	0.52	4.02	0.13	3.79	1.04	4.23	0.77	3.83	1.11	2.56	0.70	-0.71	0.02	-0.41	0.02	-0.15	—
C20:4(n-3)/ C20:3(n-6)	1.21	0.08	1.27	0.16	1.40	0.01	1.34	0.31	1.25	0.06	1.08	0.12	0.91	0.13	-0.88	8E-4	-0.69	3E-6	-0.55	6E-4
**Elongase Activities**																				
C16:0/C14:0	4.57	0.02	3.85	0.72	3.46	0.15	4.17	0.56	4.40	1.15	3.25	0.72	1.66	0.58	-0.88	8E-4	-0.70	2E-06	-0.52	0.002
C18:0/C16:0	2.62	0.04	2.58	0.29	2.47	0.25	2.57	0.44	2.57	0.23	1.99	0.24	1.41	0.54	-0.94	8E-5	-0.76	7E-8	-0.51	0.002
C20:0/C18:0	0.39	0.02	0.33	0.07	0.37	0.02	0.40	0.03	0.33	0.15	0.29	0.10	0.22	0.07	-0.86	0.002	-0.47	0.005	-0.39	0.02
